# Effect of Alum Gargles upon the Teeth

**Published:** 1883-10

**Authors:** 


### Effect of Alum Gargles Upon the Teeth.

M. Young prescribed a gargle containing a small proportion of alum for a woman suffering from chronic pharyngitis with catarrh of the middle ear. The patient, finding relief, continued its use for some three weeks. But perceiving that, at meals, her teeth began to crumble into little pieces, she consulted her dentist, who considered it due to the alum gargle, as when the enamal is removed from the teeth, the alum breaks down the dentine. To prevent this it is best, immediately after using an alum gargle, to wash the mouth out with a solution of bicarbonate of soda or an alkaline water.—*Courier Medical.*

